# Crystal structures of an ICAM-5 ectodomain fragment show electrostatic-based homophilic adhesions

**DOI:** 10.1107/S1399004714009468

**Published:** 2014-06-29

**Authors:** Rosario Recacha, David Jiménez, Li Tian, Román Barredo, Carl G. Gahmberg, José M. Casasnovas

**Affiliations:** a Centro Nacional de Biotecnología (CNB–CSIC), Campus Universidad Autónoma de Madrid, Darwin 3,, 28049 Madrid, Spain; b Division of Biochemistry and Biotechnology, University of Helsinki, 00014 Helsinki, Finland; c Neuroscience Center, University of Helsinki, 00014 Helsinki, Finland

**Keywords:** neuron, immunoglobulin superfamily, cell adhesion, ICAM-5

## Abstract

Intercellular cell adhesion molecule-5 (ICAM-5) is a member of the ICAM subfamily that is exclusively expressed in the telencephalon region of the brain. The crystal structure of the four most N-terminal glycosylated domains (D1–D4) of ICAM-5 was determined in three different space groups and the D1–D5 fragment was modelled. The structures showed a curved molecule with two pronounced interdomain bends between D2 and D3 and between D3 and D4, as well as some interdomain flexibility. In contrast to ICAM-1, ICAM-5 has patches of positive and negative electrostatic charge at D1–D2 and at D3–D5, respectively. ICAM-5 can mediate homotypic interactions. In the crystals, several charge-based intermolecular interactions between the N-terminal and C-terminal moieties of the ICAM-5 molecules were observed, which defined an interacting surface in the D1–D4 fragment. One of the crystal lattices has a molecular assembly that could represent the homophilic ICAM-5 cell adhesion complex in neurons.

## Abstract 2

Crystal structures of Intercellular Cell Adhesion Molecule-5 (ICAM-5) show charge-based homotypic interactions and a potential ICAM-5 cell adhesion complex in neurons.

## 1. Introduction   

Adhesion processes are fundamental to cell organization, communication and motility in multicellular organisms. Cell adhesion molecules participate in numerous physiological processes and specifically in blood-cell migration and immune responses (Springer, 1990 ▶). Cell–cell interactions and signalling events in the immune system resemble those between neurons in the central nervous system (CNS; Dustin & Colman, 2002 ▶; Engelhardt & Ransohoff, 2005 ▶; Gahmberg *et al.*, 2008 ▶). The ICAM subfamily of cell adhesion molecules has long been linked to adhesion processes in the immune system (Springer, 1990 ▶; Gahmberg, 1997 ▶). Characterization of ICAM-5 in neurons showed that the ICAMs are also involved in cell adhesion in the CNS (Tian *et al.*, 1997 ▶; Mizuno *et al.*, 1997 ▶). ICAM-5 is associated not only with leukocyte trafficking in the brain but also with synaptic interaction among neurons, neurite development and anti-inflammatory reactions (Gahmberg *et al.*, 2008 ▶; Ning *et al.*, 2013 ▶). ICAM-5 could be involved in brain development and synaptogenesis, since its expression begins at birth in parallel with CNS maturation and the formation of complex neural circuits; it promotes the development of neurites and dendritic filopodia (Tian, Kilgannon *et al.*, 2000 ▶; Tian, Nyman *et al.*, 2000 ▶; Furutani *et al.*, 2007 ▶; Raemaekers *et al.*, 2012 ▶). ICAM-5 association with membrane presenilin proteins links this ICAM to Alzheimer’s disease (Gahmberg *et al.*, 2008 ▶).

ICAM-5, initially called telencephalin, is an integral type I transmembrane glycoprotein of ∼150 kDa that is expressed exclusively in telencephalic neurons (Mizuno *et al.*, 1997 ▶). It has nine immunoglobulin superfamily (IgSF) domains, with a total of 832 amino acids in the extracellular region, a single transmembrane segment and a 64-residue cytoplasmic domain (Mizuno *et al.*, 1997 ▶). In addition to ICAM-5, another four major ICAM family members (ICAM-1, ICAM-2, ICAM-3 and ICAM-4) are ligands of lymphocyte function-associated antigen-1 (LFA-1; Gahmberg, 1997 ▶). They differ in their cell expression patterns and numbers of IgSF domains: ICAM-2 and ICAM-4 have two, ICAM-1 and ICAM-3 have five and ICAM-5 has nine (Gahmberg *et al.*, 1997 ▶). Domain 2 (D2) of ICAM-5 is very similar to D2 of ICAM-1 and ICAM-3 (>60% sequence identity), whereas the ICAM-5 D3–D4 sequence more closely resembles the homologous ICAM-3 domains. Like other members of the subfamily, the most membrane-distal N-terminal domain (D1) of ICAM-5 binds to the LFA-1 I-domain (Zhang *et al.*, 2008 ▶) and mediates cell adhesion interactions between T cells and neurons in the CNS (Tian, Kilgannon *et al.*, 2000 ▶). In addition to integrin recognition shared with the other ICAMs, ICAM-5 has unique characteristics within the subfamily, as it mediates homophilic adhesion interactions that promote neurite development (Tian, Nyman *et al.*, 2000 ▶). These homotypic interactions, which engage the five most N-terminal domains, are specific to ICAM-5 and might be based on electrostatic contacts between the basic (D1 and D2, pI 11.0) and acidic (D3–D5, pI 4.0) modules; this charge distribution is unique to ICAM-5.

The crystal structures of several extracellular fragments of ICAM family members show the IgSF domain conformation (Casasnovas *et al.*, 1997 ▶, 1998 ▶; Chen *et al.*, 2007 ▶), the mode of LFA-1 integrin recognition (Shimaoka *et al.*, 2003 ▶; Song *et al.*, 2005 ▶; Zhang *et al.*, 2008 ▶) and an ICAM-1 oligomer on the cell surface (Yang *et al.*, 2004 ▶). The two most membrane-distal ICAM-5 extracellular domains (D1–D2) have been crystallized in complex with the LFA-1 I-domain (Zhang *et al.*, 2008 ▶), which shows how this ICAM recognizes the integrin. The structure identified a recognition mode similar to that of other ICAMs, with a glutamic acid (Glu37) at the N-terminal domain that coordinates the metal ion at the I-domain (Zhang *et al.*, 2008 ▶). Nonetheless, there is currently no structural information on the ICAM-5 D1–D5 module that participates in homophilic adhesion interactions; it is also unclear whether this module adopts a dimeric arrangement similar to that of ICAM-1 (Chen *et al.*, 2007 ▶; Yang *et al.*, 2004 ▶). Here, we used X-ray crystallography to generate structural information on the most extracellular portion of ICAM-5. We present the crystal structure of a module containing the four most N-terminal ICAM-5 domains in three different lattices and a model of D1–D5. These structures of the ICAM-5 fragment show several charge-based intermolecular interactions, which identify a common interacting surface and a molecular arrangement that could clarify ICAM-5 homotypic cell–cell interactions in neurons.

## 2. Methods   

### 2.1. Protein expression and purification   

Recombinant ICAM-5 fragments comprising D1–D4 or D1–D5 were expressed fused to the human IgG1 Fc region in lectin-resistant CHO-Lec 3.2.8.1 cells using the glutamine synthetase system (Casasnovas *et al.*, 1997 ▶). These constructs contain the ICAM-5 cDNA encoding the indicated IgSF domains, followed by a thrombin recognition sequence, a splicing signal and the genomic DNA of the human IgG1 Fc region.

ICAM-5 D1–D4 (IC5-4D) and D1–D5 (IC5-5D) fragments were purified by multistep chromatography. After affinity chromatography using protein A Sepharose (GE), the eluted ICAM-5-Fc fusion proteins were thrombin-treated overnight at 30°C. The Fc fragment was removed with protein A Sepharose and the ICAM-5 fragment was purified by size-exclusion chromatography on a Superdex 200 column (GE) in 10 m*M* HEPES pH 7.5, 100 m*M* NaCl. Final purification of the IC5-4D and IC5-5D fragments was by anion exchange. The proteins were concentrated to 20 mg ml^−1^ for crystallization trials.

### 2.2. Crystallization and diffraction data collection   

Two crystal forms of the IC5-4D fragment were prepared at 21°C with crystallization solutions containing 10% PEG 4000 and two buffers, 100 m*M* Tris–HCl pH 8.5 for the *P*4_3_22 crystal form and 100 m*M* sodium acetate pH 5.6 for the *R*3 crystal form; these crystals diffracted to low resolution (Table 1 ▶). In addition, a few crystals smaller than those of the *P*4_3_22 form appeared in crystallization drops prepared with Tris–HCl buffer. These crystals were subsequently reproduced by microseeding in the same crystallization conditions; they belonged to space group *P*2_1_ and diffracted to ∼2.5 Å resolution (Table 1 ▶). IC5-4D crystals were dialyzed against crystallization solution with 25% ethylene glycol and flash-cooled prior to diffraction data collection on the BM16 and ID14.2 beamlines at ESRF. Diffraction data were processed with *XDS* (Kabsch, 2010 ▶) and scaled with *SCALA* from the *CCP*4 suite (Winn *et al.*, 2011 ▶). For data statistics, see Table 1 ▶.

The IC5-5D protein was crystallized using a solution consisting of 10% PEG 4000, 100 m*M* cacodylate buffer pH 7.0 and 10% 1,3-butanediol or 1,4-butanediol. The crystals were cryoprotected with crystallization solution containing 25% ethylene glycol and flash-cooled for data collection. The IC5-5D crystals diffracted to very low resolution (∼10 Å).

### 2.3. Structure determination and refinement   

The IC5-4D structure was determined from the monoclinic *P*2_1_ crystals using multiple isomorphous replacement and molecular-replacement (MR) methods. Derivative crystals were prepared by soaking in K_2_Pt(CN)_4_ and NaBr. Heavy-atom sites were determined from difference Patterson maps and were refined with *MLPHARE* from the *CCP*4 suite (Winn *et al.*, 2011 ▶), which gave a low figure of merit of 0.376. Alternatively, we applied MR with individual IgSF domains of ICAM-2 (D1; PDB entry 1zxq; Casasnovas *et al.*, 1997 ▶) and ICAM-1 (D2, D3 and D4; PDB entries 1ic1 and 1p53; Casasnovas *et al.*, 1998 ▶; Yang *et al.*, 2004 ▶). A single MR solution with the four concatenated domains was obtained using *Phaser* in *CCP*4. MR and heavy-atom phases were combined using *SIGMAA* in *CCP*4 to obtain an electron-density map. Rigid-body refinement for individual domains and Cartesian simulated annealing were initially applied for structure refinement using *PHENIX* (Adams *et al.*, 2010 ▶), which resulted in a high-quality electron-density map for manual rebuilding of the structure with *Coot* (Emsley & Cowtan, 2004 ▶). A later step consisted of refinement with *PHENIX*, applying the nonmerohedral twinning law *h*, −*k*, −*l*, which reduced the refinement statistics markedly (Table 1 ▶).

IC5-4D structures were determined at low resolution for the *P*4_3_22 and *R*3 crystals by MR using the high-resolution *P*2_1_ structure, which facilitates low-resolution structure refinement (Brunger *et al.*, 2009 ▶). In addition, we used recently implemented procedures in *phenix.refine* to improve refinement at low resolution (Headd *et al.*, 2012 ▶). After a cycle of rigid-body refinement, we applied torsion-angle dynamics simulated annealing using the MLHL target function, followed by cycles of conjugate-gradient minimization, with secondary-structure and Ramachandran plot restraints for coordinate refinement; the restrained isotropic procedure was used to refine the atomic displacement. These refinement cycles were combined with iterative rounds of manual building with *Coot* (Emsley & Cowtan, 2004 ▶). To improve the low-resolution electron-density maps, we used thermal *B*-factor sharpening, which increases the detail of side-chain conformations (Brunger *et al.*, 2009 ▶). The *P*4_3_22 crystal structure has two molecules in the asymmetric unit; its refinement included noncrystallographic symmetry restriction. Analysis of the *R*3 crystal diffraction data with *phenix.xtriage* identified one merohedral twin operator based on the *L*-test (Adams *et al.*, 2010 ▶), which was confirmed with the *SFCHECK* program in *CCP*4. The estimated twin fraction was 0.12 (Britton analysis) or 0.14 (*H*-test). The merohedral twin operator *k*, *h*, −*l* was applied in the refinement of the *R*3 crystal structure, which notably improved the process. Refinement statistics are shown in Table 1 ▶.

Coordinates and structure factors have been deposited in the Protein Data Bank as entries 4oi9 (*P*2_1_), 4oia (*P*4_3_22) and 4oib (*R*3).

### 2.4. Modelling of the ICAM-5 and ICAM-1 D1–D5 fragments and intermolecular interface analysis   

We modelled the ICAM-5 D4–D5 interdomain junction and D5 based on the ICAM-1 D3–D5 structure (PDB entry 2oz4; Chen *et al.*, 2007 ▶) using *Chimera*/*Modeller* (Pettersen *et al.*, 2004 ▶). The IC5-5D model combines the IC5-4D structure and models of the D4–D5 interdomain junction and of D5. The ICAM-1 D1–D5 model combines the D1–D2 (PDB entry 1ic1) and the D3–D5 (PDB entry 2oz4) structures based on the IC5-4D crystal structure.

Buried surfaces and residues at intermolecular contacts in the crystals were identified with the *PISA* server (http://www.ebi.ac.uk/msd-srv/prot_int/pistart.html).

## 3. Results   

### 3.1. The crystal structure of the four most N-terminal domains of ICAM-5 (IC5-4D)   

**Figure 1:**
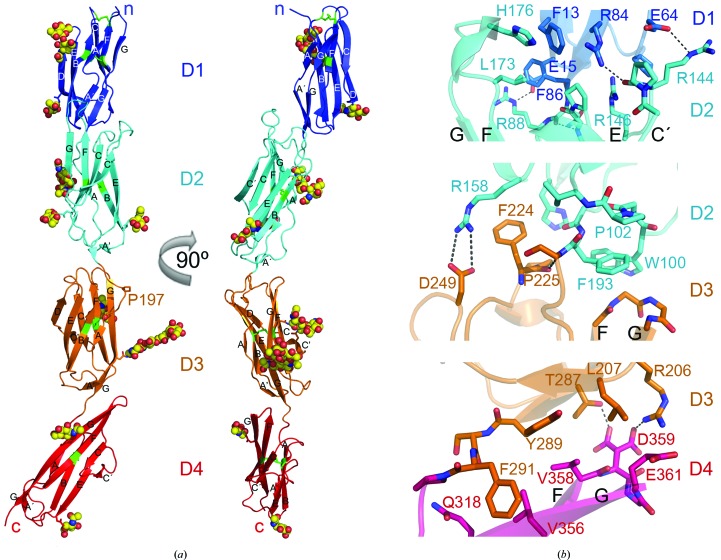
Crystal structure of the four most N-terminal domains of ICAM-5. (*a*) Ribbon diagram of the monoclinic *P*2_1_ structure with IgSF domains in blue (D1), cyan (D2), orange (D3) and red (D4). Two views are shown rotated by 90°. Side chains of asparagines in N-linked glycosylation sites are shown as sticks, and linked carbohydrates with a defined structure are depicted as spheres with carbon in yellow, nitrogen in blue and oxygen in red. Cysteines and disulfide bridges are shown in green. The β-strands, N-terminus and C-terminus are labelled. (*b*) Close-up views of the D1–D2, D2–D3 and D3–D4 interdomain junctions. Residues at the interdomain interface are shown as stick diagrams with oxygen in red and nitrogen in blue. Hydrophilic interactions are shown as dashed lines.

**Figure 2:**
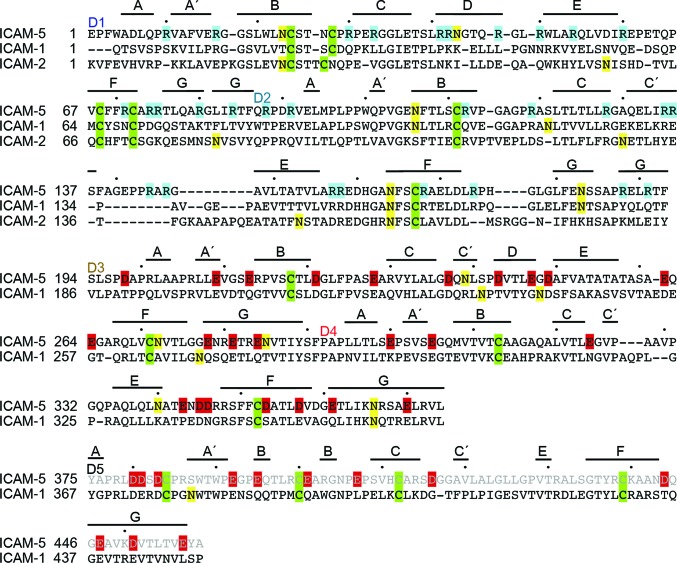
Sequence alignment of ICAM-5, ICAM-1 and ICAM-2. Structure-based sequence alignment is shown for the D1–D4 fragment and a sequence alignment is shown for D5. The ICAM-5 D1–D2 and D3–D4 modules of the monoclinic *P*2_1_ crystal structure were structurally aligned with the homologous modules of ICAM-1 and ICAM-2. The first residue of each domain is labelled and β-strands are marked above the sequences. Cysteines are highlighted in green and glycosylated Asn in yellow; basic residues in D1–D2 are in cyan and acidic residues in D3–D5 are in red.

The IC5-4D fragment is a curved molecule as a result of two sharp bends at D2–D3 (∼130° interdomain angle) and at D3–D4 (∼140° interdomain angle) (Fig. 1 ▶ *a*). The ICAM-5 D1–D2 module is more closely related structurally to ICAM-1 than to ICAM-2, with root-mean-square deviation (r.m.s.d.) of 1.75 Å for 178 residues and 2.7 Å for 174 residues, respectively. The ICAM-5 D2 sequence (66% identity) and structure are similar to ICAM-1 D2, except for the C′E loop (Supplementary Fig. S1*a* 1). In ICAM-5 the bottom of D1 contacts the C′E and FG loops at the top of D2 (Fig. 1 ▶ *b*, Supplementary Fig. S1*a*), whereas in ICAM-1 and ICAM-2 D1 only contacts the D2 FG loop (Supplementary Fig. S1*a*). His176 and Leu173 in the D2 FG loop contact Phe13 and Phe86 in D1, respectively (Fig. 1 ▶ *b*). There are also several hydrophilic interactions between D1 and D2, which engage several Arg residues. D1 and D2 are Arg-rich and have a total of 30 solvent-exposed Arg residues (Fig. 2 ▶), which are responsible for the basic pH of these domains. ICAM-5 has a relatively long, hydrophobic N-terminus (Fig. 2 ▶). There are two N-linked glycans in D1 (Figs. 1 ▶ *a* and 2 ▶); the glycan attached to Asn23 shields Trp51 from the solvent, as described for other ICAMs (Jiménez *et al.*, 2005 ▶). The N-linked glycans in ICAM-5 D2 are also found in ICAM-1: they are near the bottom of the domain (Figs. 1 ▶ *a* and 2 ▶).

The ICAM-5 D3–D4 module is structurally very similar to that of ICAM-1 (r.m.s.d. of 1.77 Å for 178 residues; Supplementary Fig. S1*b*), which correlates with their sequence similarity (∼55%). As the sequence of the ICAM-3 D3–D4 domains is even more similar to that of ICAM-5 (∼66%), the structure of these domains must also be similar. The D3–D4 module is sharply bent; D3 contacts the protruding D4 FG loop (Fig. 1 ▶) as in the ICAM-1 structure (Supplementary Fig. S1*c*). D3 and D4 belong to the I1 and I2 subsets of IgSF domains, respectively, as is the case for D1 and D2. D1 and D3 each have a D β-strand that is absent in D2 and D4 (Fig. 2 ▶). In contrast to the LFA-1-binding D1, D3 also has a short C′ β-strand (Fig. 2 ▶). At the D3 CC′ loop, ICAM-5 bears an aspartic acid residue (Asp237) which is also conserved in ICAM-1 (Asp229; Fig. 2 ▶). This residue participates in the recognition of the Mac-1 integrin by ICAM-1 (Diamond *et al.*, 1991 ▶). Although the D3 CC′ loop sequence and conformation are almost identical in both ICAM molecules (Fig. 2 ▶), ICAM-5 has two nearby glycans linked to Asn239 and Asn272 that might prevent integrin binding to the exposed Asp237 (Fig. 1 ▶ *a*). The D4 C′ edge is also unique in ICAM-1, as it has a high degree of structural flexibility and takes part in ICAM-1 dimerization (Yang *et al.*, 2004 ▶; Chen *et al.*, 2007 ▶). The C′E loop is structurally distinct in ICAM-5 (Fig. 2 ▶), and the structures reported here do not show flexibility in this region. The D4 C′ edge is involved in several crystal contacts as described below, but its conformation is preserved.

The ICAM protein crystal structures determined to date lack the D2 junction with D3. The IC5-4D structure shows that D2 tilts towards a short FG loop in D3 (Fig. 1 ▶ *a*) which bears two glycines at the tip (Fig. 1 ▶ *b*). The D3 N-terminus (Ser194–Pro197) is tangential to D3 (Fig. 1 ▶ *a*); the polypeptide kinks sharply at Pro197 and continues parallel to D4 towards β-strand A (Fig. 1 ▶ *a*). The D2 A′ β-strand is perpendicular to the D3 main axis, such that Trp100 lies on the D3 FG loop (Fig. 1 ▶ *b*). The side chain of the last D2 residue, Phe193, is enclosed by the D3 FG loop and D2 residues Trp100 and Pro102. The tilted conformation of the D2–D3 module must be forced by exposure of the bulky Phe224 at the tip of the D3 BC loop (Fig. 1 ▶ *b*). The Phe224 aromatic chain protrudes from the D3 BC loop and contacts D2 Arg158, which forms a salt bridge with D3 Asp249. All of the residues at the D2–D3 junction are conserved in ICAM-1 (Fig. 2 ▶) and ICAM-3 (not shown), indicating a conserved tilted arrangement for this module in the ICAM subfamily.

### 3.2. Interdomain mobility   

**Figure 3:**
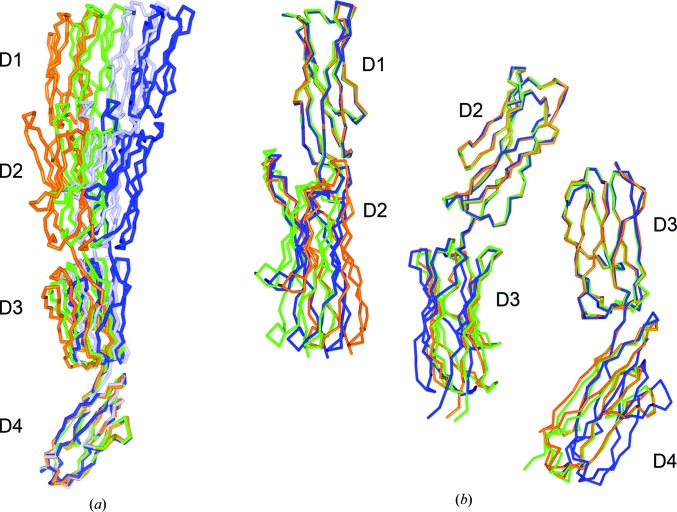
Interdomain flexibility in ICAM-5. Superposition of the IC5-4D structure determined in three space groups: *R*3, orange; *P*2_1_, green; *P*4_3_22, light blue (molecule *A*) and dark blue (molecule *B*). Superposition of the IC5-4D structures based on D4 (*a*) or superposition of two domain modules (*b*).

Comparison of the three crystal forms indicated some flexibility at the interdomain interfaces (Fig. 3 ▶), which is probably needed to facilitate cell–cell interactions. Superposition based on D4 showed that the IC5-4D molecules moved in a single direction (Fig. 3 ▶ *a*). The largest movement between the most divergent *R*3 and *P*4_3_22 structures (r.m.s.d. of 2.7 Å) was ∼16°. The overall *P*2_1_ structure is nonetheless more similar to the *R*3 crystal structure (5°) than to the *P*4_3_22 structure (11°), even though the tetragonal and monoclinic crystals were prepared under the same conditions. This could indicate that interdomain movement depends more on crystal contacts than on crystallization conditions. The interdomain mobility in the two-domain modules is relatively similar (12° for D1–D2, 10° for D2–D3 and 15° for D3–D4; Fig. 3 ▶ *b*). The D1–D2 module conformation is distinct in the three structures, whereas the D2–D3 and the D3–D4 modules adopt similar conformations in the *P*2_1_ and *R*3 structures (Fig. 3 ▶ *b*).

### 3.3. Overall structures of the D1–D5 fragments of ICAM-5 and ICAM-1   

**Figure 4:**
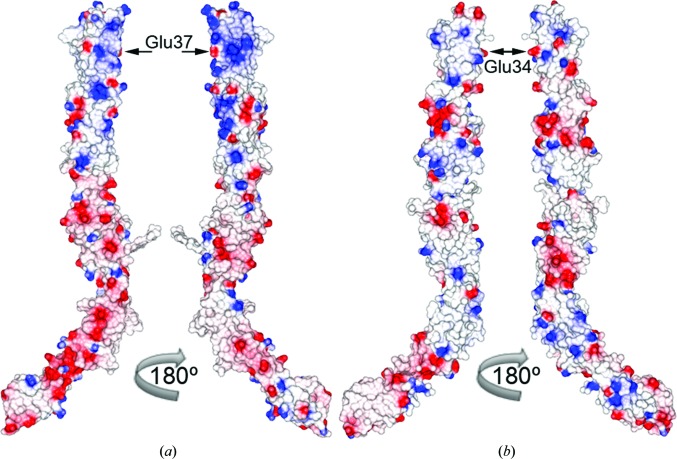
Electrostatic surface potential of the five N-terminal domain fragments of ICAM-5 (*a*) and ICAM-1 (*b*). Surface representations are shown in two orientations. The central LFA-1 integrin-binding residues in D1 are marked with arrows.

The ICAM-5 D1–D5 fragment engages in homotypic interactions (Tian, Nyman *et al.*, 2000 ▶). The low-resolution diffraction of the IC5-5D crystals nonetheless hindered structure determination. We used homology modelling of D5 to generate the complete structure of the D1–D5 fragment (Supplementary Fig. S2*a*). Modelling was based on the ICAM-1 D3–D5 structure (Chen *et al.*, 2007 ▶). The D5 domains of ICAM-1 and ICAM-5 are very similar (49% sequence identity), and the D4–D5 junction preserves the ICAM-1 residues that participate in interdomain interactions (Supplementary Fig. S2*b*). We similarly modelled the ICAM-1 D2–D3 junction based on the IC5-4D structure, which provided a complete view of the extracellular portion of ICAM-1 (Supplementary Fig. S2 ▶ *a*, green). The overall shape of the five extracellular N-terminal domains of ICAM-1 and ICAM-5 is preserved (Fig. 4 ▶). The molecules are curved owing to the bent conformation of the D2–D3 and D3–D4 modules. D5 is not heavily glycosylated in either molecule (Fig. 2 ▶). In ICAM-1 glycans accumulate at D2 and the top of D3, whereas in ICAM-5 the glycans are distributed more evenly from D1 to D5 (Supplementary Fig. S2a). The CFG face of D3 is heavily glycosylated in ICAM-5 (Fig. 1 ▶ *a*).

A major difference between the D1–D5 fragments of ICAM-1 and ICAM-5 is their electrostatic potential. Compared with other ICAMs, ICAM-5 has an unusual charge distribution, with D1 and D2 being highly basic (pI ∼11) and D3–D5 being acidic (pI ∼4). ICAM-1 does not show this marked difference between the N-terminal and C-terminal modules, and the IgSF domains have varied pI values of 7.8, 6.1, 4.0, 9.0 and 5.1 for D1, D2, D3, D4 and D5, respectively. The structures of all five ICAM-1 and ICAM-5 domains thus had very distinct charge distributions (Fig. 4 ▶). ICAM-5 D1 and D2 had large patches of positive density (Fig. 4 ▶), owing to the large number of arginines (Fig. 2 ▶), which surrounded the integrin-binding Glu37 in D1 and were found in the CFG β-sheet and in β-strand D. Some of these arginines bind to the LFA-1 I-domain (Zhang *et al.*, 2008 ▶). The tip of D1 was also basic (Fig. 4 ▶), with Arg residues in the BC, DE and FG loops (Fig. 2 ▶). Acidic residues prevailed in ICAM-5 D3–D5 (Fig. 4 ▶), and were scattered from D3 to the top of D5 and enriched on one side of the molecule. The Mac-1 integrin-binding D3 of ICAM-1 also has a negatively charged patch, but D4 was less acidic in ICAM-1 than in ICAM-5 (Fig. 4 ▶).

### 3.4. Electrostatic-based homotypic ICAM-5 interactions in the crystals   

**Figure 5:**
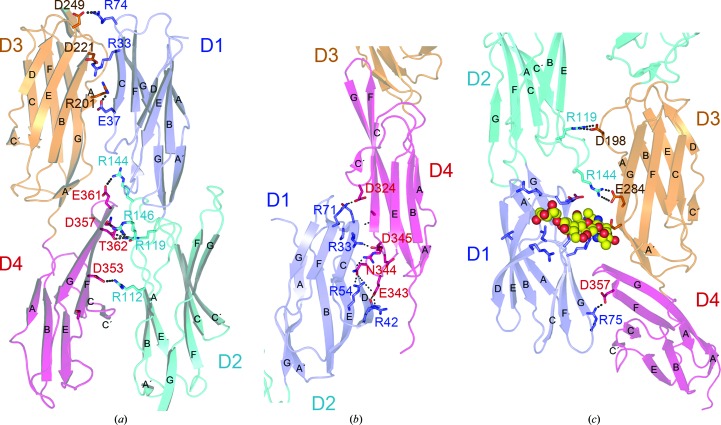
Electrostatic-based intermolecular interactions between N-terminal and C-terminal modules of IC5-4D in the crystals. Ribbon diagram of interacting domains colored as in Fig.1 ▶ (see also Supplementary Fig. S3). β-strands and interacting charged residues, shown in Supplementary Table S1, are labeled. Salt bridges and hydrogen bonds are shown as dashed lines. (*a*) Contact in the *P*4_3_22 crystal structure (interface A). (*b*) Contact in the *P*2_1_ crystals (interface B), also observed in the *R*3 crystal structure. (*c*) Contact in the *P*2_1_ crystal structure (interface C). The glycan linked to Asn282 is as spheres with carbon in yellow, nitrogen in blue and oxygen in red.

Homophilic ICAM-5 adhesions are mediated by interaction of the basic N-terminal D1–D2 with the acidic D3–D5 (Tian, Nyman *et al.*, 2000 ▶). Our IC5-4D crystal structures showed several types of interactions between the N-terminal and C-terminal portions of symmetry-related molecules which involved charged residues (Fig. 5 ▶, Supplementary Table S1). Based on buried surface area (BSA), the most stable of these interactions was in the *P*4_3_22 crystal structure (interface A) between two symmetry-related IC5-4D molecules (Fig. 5 ▶ *a*). This interaction between the N-terminal and C-terminal two-domain modules buried ∼830 Å^2^ of each molecule, including charged residues in the IgSF domains (Supplementary Table S1). The molecules follow a head-to-tail parallel packing arrangement (Supplementary Fig. S3*a*). A charged region on the D1 CFG face, which includes the integrin-binding Glu37, interacts with the negatively charged D3 (Fig. 5 ▶ *a*, Supplementary Fig. S3*a*). The *PISA* server identified six salt bridges between interacting molecules, three in the D1/D3 interface and four in the D2/D4 interface (Fig. 5 ▶ *a*, Supplementary Table S1); positively charged D1–D2 regions were completely buried by the negatively charged D3–D4 regions (Supplementary Fig. S3*a*). The *P*2_1_ and *R*3 crystal structures also show head-to-tail intermolecular contacts (interface B) and a parallel arrangement of the IC5-4D molecules (Fig. 5 ▶ *b*, Supplementary Fig. S3*b*). These contacts connect D1 and D4 from two symmetry-related molecules and bury ∼600 Å^2^ of surface area in the *P*2_1_ crystal contact. Interface B includes positively and negatively charged surfaces in D1 and D4, respectively, and four salt bridges (Supplementary Table 1 ▶, Supplementary Fig. S3*b*).

In the *P*2_1_ crystal structure, we identified two similar antiparallel arrangements of symmetry-related ICAM-5 molecules with contacts between the N-terminal and C-terminal portions (Supplementary Figs. S3*c* and S3*d*). The head (D1–D2) and tail (D3–D4) of one IC5-4D molecule contacted two distinct symmetry-related partners; each contact buried 500–600 Å^2^ of surface area and included charged residues in all four interacting domains. The first contacts, with the largest interface (600 Å^2^ BSA), were formed by D1–D2 binding to D3 and the top half of D4 (interface C; Fig. 5 ▶ *c*); a glycan contributed 50% of the buried surface. Three of the glycan carbohydrates attached to Asn285 in D3 were well defined in the structure and established an extended interaction network with the bottom of D1 (Fig. 5 ▶ *c*, Supplementary Table S1). In addition, three acidic residues in D3–D4 formed salt bridges with the D1–D2 arginines. The second type of crystal contact buried a smaller surface area (500 Å^2^) and included no glycans (interface D; Supplementary Fig. S3*d*); a single IC5-4D molecule also interacted with two symmetry-related molecules that buried about 1000 Å^2^ of its surface. D1–D2 contacted D4 and involved charged residues (Supplementary Table S1).

### 3.5. ICAM-5 surfaces that mediate homotypic interactions   

**Figure 6:**
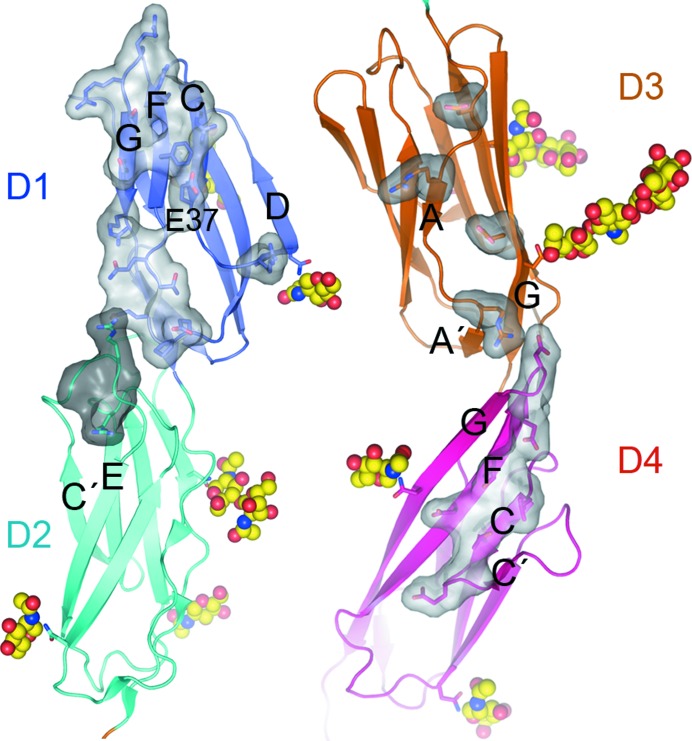
ICAM-5 surfaces that mediate homotypic interactions. Ribbon diagram of the D1–D2 (left) and D3–D4 (right) modules, with residues involved in intermolecular contacts in several crystals shown as sticks with surfaces in grey. These residues are buried in at least two of the three interfaces in Fig. 5 ▶, which are representative of the three crystal structures. Domain regions with buried residues are labelled, as is the LFA-1 integrin-binding residue Glu37.

The intermolecular contacts in the ICAM-5 crystals can help to identify surfaces involved in homophilic adhesions (Tian, Nyman *et al.*, 2000 ▶). Interfaces A and C were formed by D1–D2 binding to D3–D4 in two distinct crystal forms, with the interacting molecules adopting distinct orientations (Fig. 5 ▶). In interface B, D1 interacted similarly with D4 in the *P*2_1_ and *R*3 crystal structures. We identified shared residues in these interfaces (Fig. 6 ▶). A D1 region involved in LFA-1 integrin binding, which surrounds β-strand C and Glu37 (Zhang *et al.*, 2008 ▶), was partially buried in several interfaces (Figs. 5 ▶ and 6 ▶). The ICAM-5 I-domain-binding region includes residues in the CFG β-sheet and the CD edge of the domain (Zhang *et al.*, 2008 ▶). Interfaces A and C mainly included residues in the CFG β-sheet, whereas interface B also covered the CD edge of D1 (Fig. 5 ▶). Most D1 residues that bound to D3–D4 extended from the top to the bottom of the CFG β-sheet (Fig. 6 ▶). At the top of D2, near D1, Arg119 and Arg144 at the C′ edge of the domain contacted D3–D4 in interfaces A and C (Figs. 5 ▶ and 6 ▶).

The few D3 residues that bound to other molecules in several crystals were scattered on the same side of the D3 domain and of the D4 surface that mediates interactions (Fig. 6 ▶). This D4 surface covered the upper half of the CFG β-sheet. The interacting surfaces of D1 and D4 were broader than those of D2 and D3, and also included the CFG β-sheet, which is commonly involved in cell adhesion interactions by IgSF members (Wang, 2002 ▶). The large interacting surfaces in D1 and D4 suggest that they play an important role in ICAM-5 homotypic interactions.

## 4. Discussion   

Using X-ray crystallography and homology modelling, we determined the structure of the five most N-terminal domains of ICAM-5. The crystal structures show an I1–I2–I1–I2 fold for the ICAM-5 D1–D4 module and certain interdomain flexibility, which could be necessary for cell adhesion interactions. The ICAM-5 D1–D5 fragment has a curved structure, with two pronounced bends between D2–D3 and D3–D4. The D3–D4 conformation resembles that reported for the same module of ICAM-1 (Yang *et al.*, 2004 ▶). Here, we show that the D2–D3 module is also bent. Given the great resemblance of their interdomain interfaces, other ICAMs are likely to have a similar conformation for this module. The relatively conserved five-domain extracellular fragments of ICAM-1, ICAM-3 and ICAM-5 might thus adopt a similar S-shaped configuration in all three molecules.

Binding studies using various ICAM-5 domain fragments showed that homotypic interactions are mediated by the D1–D5 fragment (Tian, Nyman *et al.*, 2000 ▶). The D1–D2 fragment binds weakly to ICAM-5 domain variants that contain the three most N-terminal domains (D1, D1–D2 or D1–D3) and more strongly to longer variants that also contain D4 or D5 (D1–D4 or D1–D5; Tian, Nyman *et al.*, 2000 ▶). The binding of D1–D2 to proteins bearing D1–D4, D1–D5 or D1–D9 was similar, indicating that D4 is critical for ICAM-5 homotypic inter­actions between the N-terminal and C-terminal portions of the D1–D5 fragment. Antibody blocking experiments showed that D1 is also critical (Tian, Kilgannon *et al.*, 2000 ▶). The crystal structures reported here show several types of interactions between D1–D2 and D3–D4, and define a protein surface that can engage in homotypic ICAM-5 interactions. This surface is particularly broad in D1 and D4, both of which have a critical role in ICAM-5 homophilic adhesions (Tian, Nyman *et al.*, 2000 ▶). The D1 surface overlaps the integrin-binding region, which is positively charged in ICAM-5 and can thus interact with exposed, negatively charged regions in D4 or in the D3–D5 fragment. These structural findings further demonstrate the function of the N-terminal portion of ICAM-5 in homotypic interactions (Tian, Nyman *et al.*, 2000 ▶) and show the electrostatic nature of these interactions and the involvement of the integrin-binding region.

**Figure 7:**
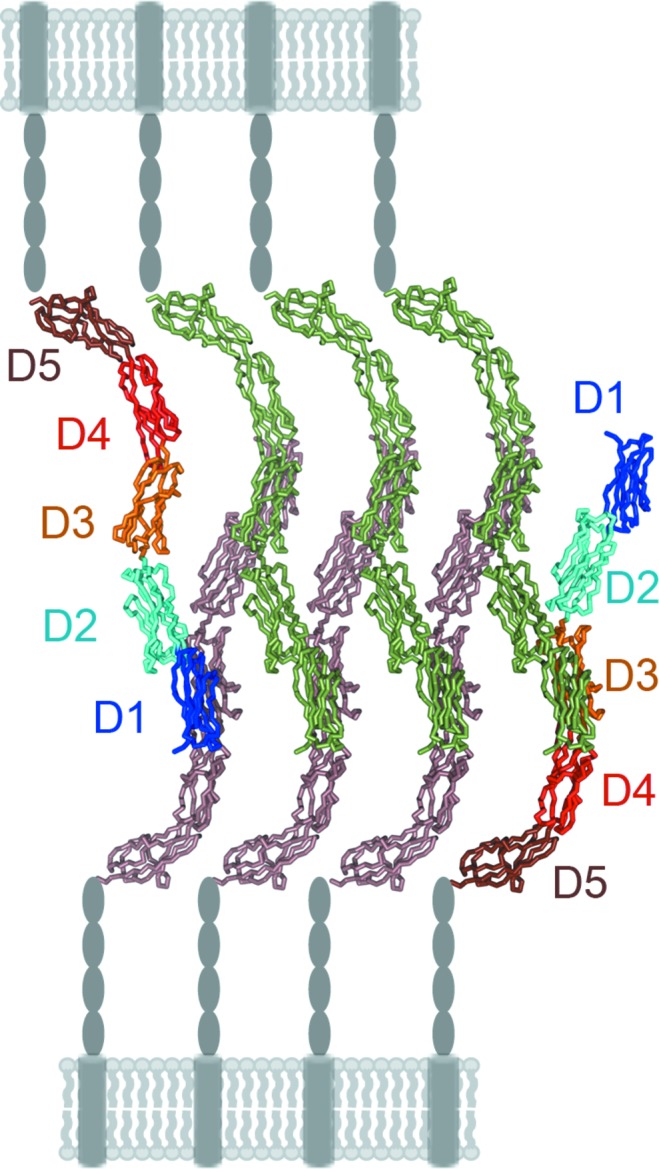
Molecular model of the ICAM-5 homophilic cell adhesion complex. Two sets of ICAM-5 molecules from two different cells interact and form a *trans*/*trans* zipper adhesive structure (Aricescu & Jones, 2007 ▶). The complex was generated in the monoclinic crystal lattice by molecules assembled as in Supplementary Fig. 3(*c*). Molecules at the edges of the zipper are shown with domains D1–D5 coloured as in Fig. 1 ▶, whereas the other molecules are green or brown. The ICAM-5 extracellular (D6–D9, ovals) and transmembrane (cylinder) domains and the cell surface are shown in grey.

Cell adhesion molecules of the IgSF can form zipper-like assemblies at cell–cell contacts which are necessary to mediate tight adhesions by low-affinity individual electrostatic interactions (Aricescu & Jones, 2007 ▶). The IC5-4D structures showed several molecular assemblies formed by symmetry-related molecules in the crystals (Supplementary Fig. S3). Some are configured by a parallel array of molecules that are not representative of interactions between molecules located on different cells (Supplementary Figs. S3*a* and S3*b*). In contrast, in the monoclinic *P*2_1_ crystals the IC5-4D molecules assemble with an antiparallel orientation (Supplementary Figs. S3*c* and S3*d*) and the crystal lattice generates two similar antiparallel *trans*/*trans* zippers (one of which is shown in Fig. 7 ▶) that resemble the assemblies described for other homophilic cell adhesion structures (Aricescu & Jones, 2007 ▶). This type of zipper could represent of ICAM-5 homophilic cell adhesion complexes. The overall curved conformation and interdomain flexibility in the extracellular portion of the ICAM proteins could facilitate zipper formation.

Our ICAM-5 crystal structures nonetheless do not include D5, so that the local contacts could differ from those shown by the IC5-4D structures. In the *trans*/*trans* zippers, each ICAM-5 molecule contacts two molecules on the membrane of a distinct cell (Fig. 7 ▶), which resembles the way ICAM-1 oligomerizes on the cell surface. The ICAM-1 *cis* oligomers are built by contacts between N-terminal modules (D1–D2) and between C-terminal modules (D4–D5) of two different molecules (Yang *et al.*, 2004 ▶). The curved ICAM-1 structure is necessary for contact with two neighbouring molecules and the formation of W-shaped ICAM-1 tetramers. It is thus likely that similar homotypic interaction modes are used to build adhesion structures on the surface of a single cell in the case of ICAM-1 or of two different cells for ICAM-5.

ICAM subfamily members share a distinctive integrin-binding surface for recognition of the integrin LFA-1 I-domain (Casasnovas *et al.*, 1997 ▶; Shimaoka *et al.*, 2003 ▶; Song *et al.*, 2005 ▶; Zhang *et al.*, 2008 ▶). IgSF domain folding and interdomain arrangement are conserved in the ICAMs, which nonetheless differ in tissue distribution, integrin binding affinity and oligomerization on the cell surface. Some ICAM proteins can also mediate molecule-specific interactions, such as ICAM-1 and ICAM-5 binding to Mac-1 and α5β1 integrins, respectively, or the homophilic adhesions described for ICAM-5 (Diamond *et al.*, 1991 ▶; Tian, Nyman *et al.*, 2000 ▶; Ning *et al.*, 2013 ▶). Some of these differences in ICAM ligand-binding activity are associated with a divergence of glycan distribution in extracellular regions. The lack of a highly conserved N-linked glycan in the ICAM-1 N-terminal domain permits the recognition of human rhinovirus, as well as ICAM-1 dimerization on cell surfaces (Casasnovas *et al.*, 1998 ▶; Yang *et al.*, 2004 ▶; Jiménez *et al.*, 2005 ▶). In a similar manner, the Mac-1 integrin-binding aspartic acid in ICAM-1 D3 is more accessible to ligands than the same residue in ICAM-5 because of the absence of glycans. The ICAM-5 crystal structure also shows distinct charged surfaces in its N-terminal (D1–D2) and C-terminal (D3–D5) fragments, which are involved in intermolecular interactions, some of which could mediate ICAM-5-specific homophilic adhesions between neurons. These results extend our understanding of the ICAM subfamily and show that the charge distribution and glycosylation of ICAM extracellular regions are responsible for the specific functions described for some members of this family of cell adhesion molecules.

## Supplementary Material

Supplementary Table 1, Supplementary Figure 1, Supplementary Figure 2 and Supplementary Figure 3.. DOI: 10.1107/S1399004714009468/yt5069sup1.pdf

PDB reference: ICAM-5 D1–D4, 4oi9

PDB reference: 4oib

PDB reference: 4oia

## Figures and Tables

**Table 1:** Data-collection and refinement statistics The *P*4_3_22 crystal structure contains two independent IC5-4D molecules in the asymmetric unit. Values in parentheses are for the highest resolution shell.

Data processing			
Space group	*P*4_3_22	*R*3	*P*2_1_
Unit-cell parameters			
*a* (Å)	96.07	228.56	76.59
*b* (Å)	96.07	228.56	46.91
*c* (Å)	321.92	69.98	95.79
α (°)	90	90	900
β (°)	90	90	104.3
γ (°)	90	120	90
Wavelength (Å)	0.97914	0.97914	0.97934
Resolution (Å)	25–3.7 (3.90–3.70)	25–3.7 (3.90–3.70)	25–2.5 (2.64–2.50)
Unique reflections	16927	14474	23146
*R*_sym_ or *R*_merge_ (%)	13.1 (38.0)	7.9 (79.6)	6.3 (34.3)
〈*I*/σ(*I*)〉	5.2 (2.0)	7.7 (1.0)	7.7 (2.2)
Completeness (%)	99.6 (99.6)	99.9 (100)	99.7 (100)
Multiplicity	7.4 (7.8)	5.8 (5.8)	3.7 (3.8)
Refinement			
Resolution (Å)	25–3.7	25–3.7	25–2.5
No. of reflections	16867	11463	23137
*R*_work_/*R*_free_ (%)	23.0/28.0	22.9/27.3	23.9/26.6
No. of atoms			
Protein	5704	2827	2912
Carbohydrates	397	284	204
Ligands	4		124
Water			94
Average *B* factors (Å^2^)			
Protein	140	185	84
Carbohydrates	181	268	112
Ligands	94		44
Water			57
R.m.s. deviations			
Bond lengths (Å)	0.003	0.005	0.004
Bond angles (°)	0.734	1.105	0.967

## Footnotes

1 Supporting information has been deposited in the IUCr electronic archive (Reference: YT5069).
