# Supplementary material for: Crystal structures of an ICAM-5 ectodomain fragment show electrostatic-based homophilic adhesions
Source: Acta Crystallogr D Biol Crystallogr. 2014 Jun 29;70(Pt 7):1934–43. doi: 10.1107/S1399004714009468 (PMC4089487; doi:10.1107/S1399004714009468)
Supplement: Supplementary file 1 [file d-70-01934-sup1.pdf]

Supplementary Table 1. Interactions between the N- (D1-D2) and C- (D3-D4) terminal modules of the IC5-4D molecule in the crystals.

Interface A: $P4_322$ (840 Å^2)

D1	Arg 33	Asp 221	D3
	Glu 37	Arg 201	
	Arg 74	Asp 249	
D2	Arg 112	Asp 353	D4
	Arg 119	Asp 357	
		Thr 362	
	Arg 144	Glu 361	
	Arg 146	Thr 362	

Interface B: $P2_1$ (570 Å^2), R3 (470 Å^2)

D1	Glu 32	Ser 349	D4
	Arg 33	Asp 345	
		Asn344	
	Leu 36	Asn 344	
	Arg 42	Glu 343	
	Thr 45	Glu 343	
	Arg 47	Tyr375	
	Gly48	Arg347	
	Arg 54	Glu 343	
		Asn 344	
	Arg 71	Glu 324	

Interface C: $P2_1$ (600 Å^2)

D1	Arg 75	Asp 357	D4
D2	Arg 119	Asp 198	D3
	Arg 144	Glu 284	
D3	NAG 2851	Ser 39	D1 D2
		Glu 61	
		Glu 63	
		Thr64	
		Gln 65	
		Arg 80	
		Arg 144	
	NAG 2852	Ser39	D1
		Glu61	
		Glu63	
		Thr64	
	BMA 2853	Ser 39	D1
		Leu 40	
		Arg 41	
	MAN 2854	Leu 40	D1
		Arg 41	
		Val 57	
		Asp 58	
		Arg 60	
		Glu 61	

Interface D: $P2_1$ (500 Å^2)

D1	Glu 15	Arg 372	D4
		Tyr 375	
	Ser 19	Ser 304	
D2	Arg 88	Asp 346	
		Val 373	
	Asp 90	Arg 372	

Intermolecular interfaces A to D in the IC5-4D crystals, with buried surface areas (parenthesis) over 500 Å^2 in each interacting molecule. Residues involved in polar interactions (salt bridges and hydrogen bonds) between IC5-4D molecules, computed with PISA. Residues in the N-terminal D1-D2 module and the interacting residues in the C-terminal D3-D4 module are on the same file of the table. Basic and acid residues are in blue and red, respectively. Interacting molecules are shown in Supplementary Figure 3.

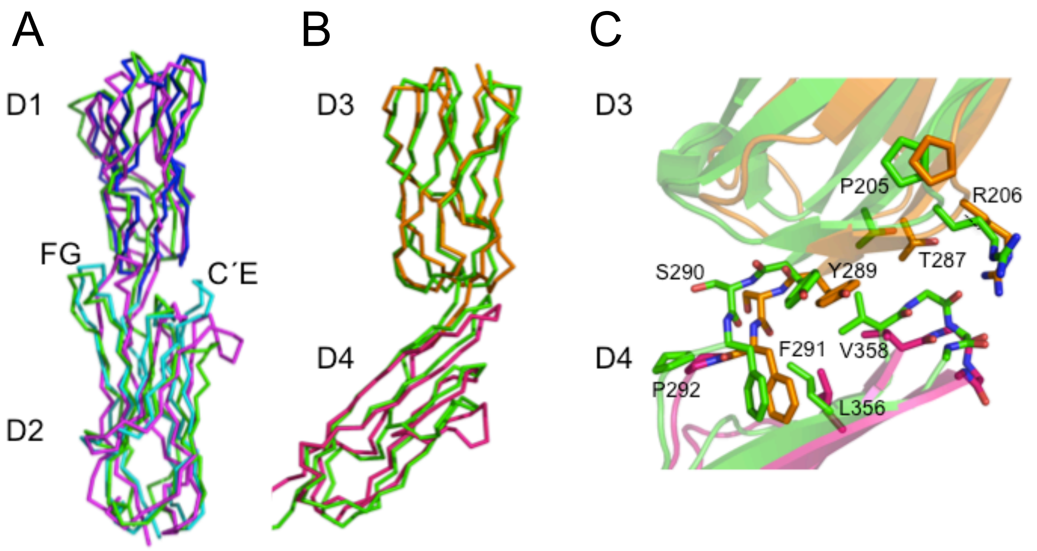

Supplementary Figure 1. Two-domain modules in the ICAM subfamily. (A) Superposition of the D1-D2 modules of ICAM-5 (D1 blue and D2 cyan), ICAM-1 (green) and ICAM-2 (magenta). (B) Superposition of the D3-D4 modules of ICAM-5 (D3 orange and D4 red) and ICAM-1 (green). (C) View of the D3-D4 interdomain region in ICAM-5 and ICAM-1, with conserved residues shown in stick. ICAM-5 residues labeled.

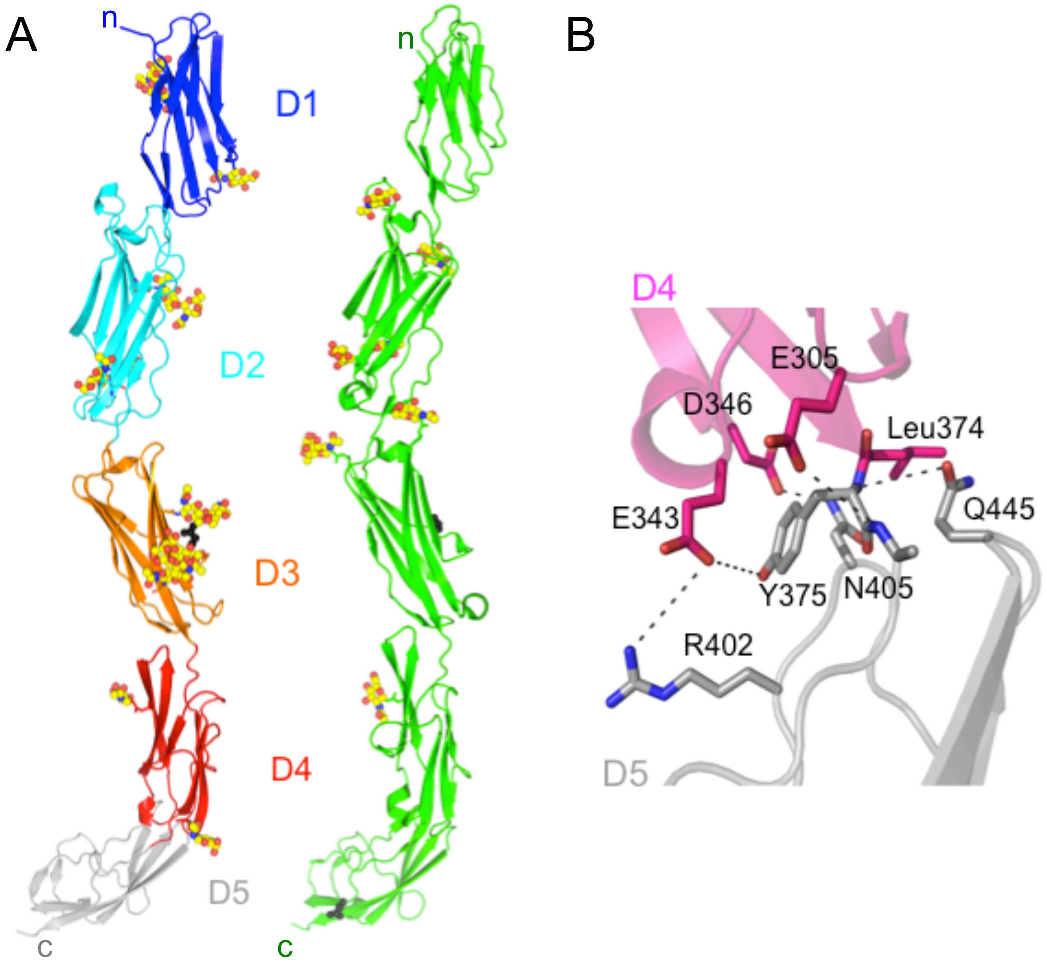

Supplementary Figure 2. Structural models of the N-terminal five domain fragments of ICAM-5 and ICAM-1. Ribbon diagrams of models prepared as described in Methods. (A) The IC5-5D fragment is shown on the left with D1-D4 fragment structure represented in Figure 1, and the modeled D5 in grey. The ICAM-1 structure is green. Side chains of asparagines with N-linked glycosylations are shown in sticks, and the N-linked carbohydrates in the structures as spheres with carbon in yellow, nitrogen, blue and oxygen, red. Asparagine residues without any modeled carbohydrate in the structures (Asn239 in ICAM-5; Asn233 and Asn379 in ICAM-1) are shown in black. (B) View of the modeled D4-D5 interdomain region of ICAM-5, with conserved residues in ICAM-1 shown in stick.

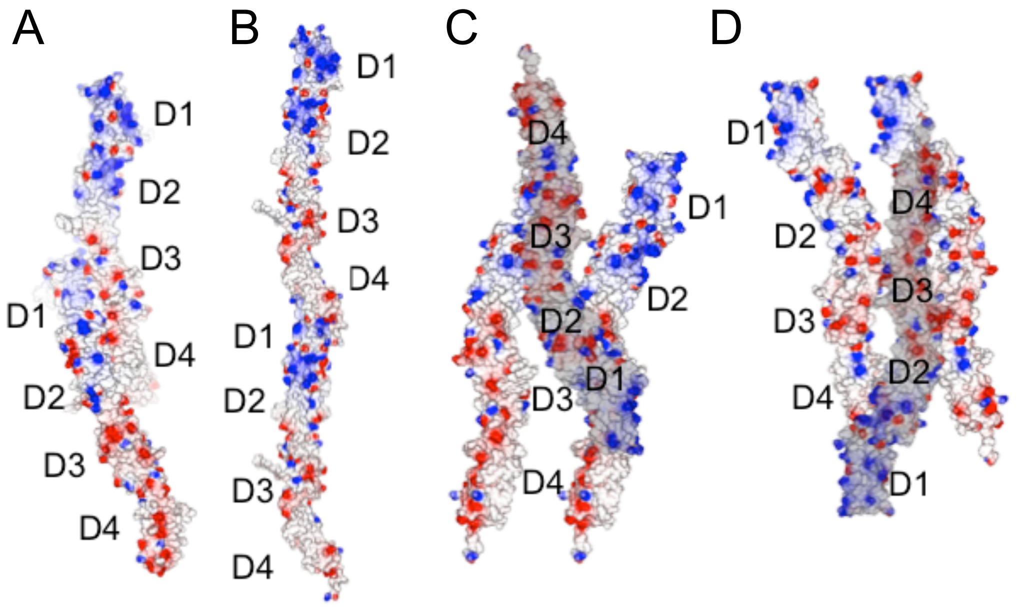

Supplementary Figure 3. Interactions between the N and C-terminal portions of IC5-4D molecules in the crystals. Surface representation of interacting IC5-4D molecules showed with their electrostatic surface potential. Homodimers with the highest buried surface area (BSA $\geq$ 500 Å^2) in the IC5-4D crystal structures are show. Intermolecular interaction in the P4$_3$22 (interface A) and P2$_1$ (interface B) crystal structures are shown in (A) and (B), respectively, where molecules follow head-tail parallel packing. Homodimers shown in (B) are similar to crystal contacts found in the R3 crystal structure. Two distinct antiparallel arrangements of the IC5-4D molecules in the P2$_1$ crystal structure, interfaces C and D, are shown in (C) and (D), respectively. The head (D1-D2) and tail (D3-D4) of one IC5-4D molecule (shaded) contacts two symmetry-related molecules, so that ~1000 Å^2 of its accessible surface is buried. Buried surface areas and residues in the interfaces are included in Supplementary Table 1. Details of the interfaces A to C are shown in Figure 5.
